# Effectiveness of Bioenergy Economy-based Health Improvement (BEHI) versus Acceptance and Commitment Therapy (ACT) on the Psychological Comorbidities and Quality of Life in Patients with Inflammatory Bowel Disease

**DOI:** 10.1093/crocol/otaf018

**Published:** 2025-03-25

**Authors:** Farzad Goli, Afsoon Derakhshanjan, Sarvenaz Jahanzad, Seyyed Abbas Haghayegh, Hamid Afshar Zanjani, Peyman Adibi

**Affiliations:** The Iranian Academy of Medical Sciences, Tehran, Iran & Behi Academy, BC, Canada; General Psychology, Department of Psychology, Najafabad Branch, Islamic Azad University, Najafabad, Iran; Cognitive Psychology, Department of Psychology, Georgia Institute of Technology, GA, USA; Department of Psychology, Najafabad Branch, Islamic Azad University, Najafabad, Iran; Department of Psychiatry, School of Medicine and Psychosomatic Research Center, Khorshid Educational and Medical Center, Isfahan University of Medical Sciences, Isfahan; Isfahan Gastroenterology and Liver Research Center, Isfahan University of Medical Sciences, Isfahan, Iran. adibi@med.mui.ac.iR

**Keywords:** acceptance and commitment therapy, quality of life, inflammatory bowel diseases

## Abstract

**Background:**

Inflammatory IBD has a significant adverse influence on the physical, psychological, family, and social dimensions of patients. This research aims to compare the effectiveness of ACT and BEHI on perceived stress, quality of life, sense of coherence, and D personality type in patients with IBD.

**Methods:**

This study used a quasi-experimental method with a pre-test, post-test, and follow-up design with a control group. A total of 37 patients were randomly selected based on a random allocation system. Data were collected by WHOQOL-BREF, the Sense of Coherence (SOC) questionnaire, the Type D Personality Questionnaire, and the Perceived Stress Scale (Perceived Stress Scale (PSS)). The first experimental group (*n* = 12) underwent ACT within eight 90-minute weekly sessions, which lasted eight weeks. The second group (*n* = 12) experienced bio-energy economy within eight 90-minute weekly sessions, lasting for eight weeks. The control group (*n* = 13) received no training in this period. Data was analyzed using a mixed variance analysis method.

**Results:**

Results revealed that perceived stress, quality of life, sense of coherence, and D personality type significantly improved in the post-test and follow-up stages through the ACT and BEHI programs (*P* < .01). It was found that there was a significant difference between the experimental groups and the control group over time (*P* < .01).

**Conclusions:**

It can be concluded the BEHI program was more effective in decreasing perceived stress and D personality type and increasing quality of life than the ACT program. Although the BEHI program improved a sense of coherence, the ACT program was more effective in increasing the sense of coherence in patients.

## Graphical Abstract

**Graphical Abstract:**
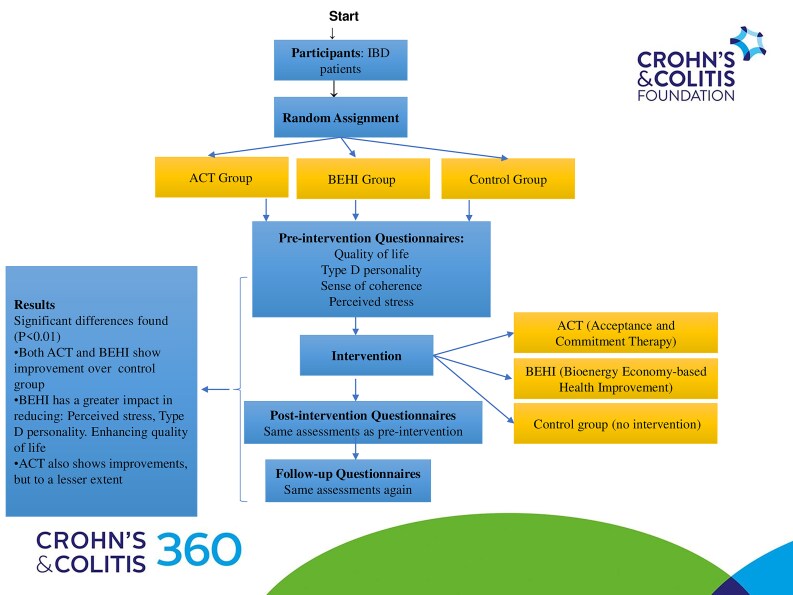

## Introduction

Inflammatory IBDs that are chronic and relapsing result from a reaction to gut microbes in individuals with genetic susceptibility.^1^ Individuals diagnosed with Inflammatory Bowel Disease (IBD) often face challenges that affect not only their physical health but also their mental, familial, and social aspects.^2^ Clinicians and patients have long suspected a connection, between psychological factors and the worsening of symptoms in individuals, with IBD.^3^

There appears to be a connection discovered linking stress to increased disease activity, in Inflammatory Bowel Disease (IBD). Research has shown that experiencing challenging life situations, stress, depression, and having a Type D personality can greatly raise the chances of a relapse, in individuals, with IBD.^4^

In terms of mental comorbidity, high levels of stress have been linked to Inflammatory Bowel Disease (IBD) resulting in a decline, in overall quality of life and increased mental health issues.^5^ The way a patient responds psychologically and the result they experience could also be influenced by variations showing that psychological mechanisms can have an impact, on this aspect.^6^ Personality type D is one feature that IBD patients tend to exhibit,^7^ and it can lower these patients’ quality of life.^8^ Type D personality is a tendency to have increased levels of negative emotions and a simultaneous tendency to suppress these emotions in social interactions because of fear of rejection or disapproval. This personality type has 2 main components. Negative Affectivity is a tendency to experience negative emotions such as worry, tension, and unhappiness across time and situations. Social inhibition is the avoidance of expressing emotions in social settings because of apprehension about disapproval or rejection.^9^ A study involving 2275 IBD patients found that 29.5% exhibited Type D personality traits. This group was significantly more likely to experience depressive symptoms and had a higher risk of future active disease. Notably, the combination of Type D personality and depressive symptoms further increased the risk for active disease.^10^ In addition to personality type and stress, Sense of Coherence (SOC) has been identified as a major, albeit indirect, component that can impact psychological distress and various health-related quality of life (HRQoL) categories in individuals with IBD. SOC, a notion that promotes health and reflects an individual’s reaction to stressful events, may be essential for managing a chronic condition.^11^ Feritas et al. discovered that decreased SOC was independently linked to greater levels of anxiety and sadness in IBD patients.^12^

Patients with IBD who had psychiatric comorbidities experienced negative illness impressions, poor medication adherence, sleep problems, and abdominal pain.^13^ Research indicates that over 40% of patients discontinue their drugs regularly, and 33% of patients still experience symptoms. One of the issues that has compelled researchers to search for alternative strategies for symptom management and psychological advancement is these restrictions.^3^ More data than ever suggests the gut–brain axis’s impact on the course and management of inflammatory Inflammatory Bowel Disease (IBD). Several systematic reviews and meta-analyses demonstrate the beneficial effects of different psychological therapies, including solution-focused, CBT, psychodynamic, and mindfulness-based therapies^14–16^

Acceptance and Commitment Therapy (ACT) is a common behavioral approach to health and illness that can be used as a successful intervention to help people with IBD feel less distressed, have a higher quality of life, and exhibit fewer type D personality traits.^17^ Instead of trying to alter or lessen unpleasant and undesired internal experiences—such as stress, unwanted thoughts, and emotions—ACT seeks to increase psychological flexibility and develop strategies for reducing the impact of these experiences on day-to-day functioning and goal fulfillment.^18^ Reduced psychological anguish in crippling chronic diseases is linked to the psychological flexibility provided by ACT.^19^

Equivalent to Acceptance and Commitment Therapy (ACT), bioenergy economy-based health improvement (BEHI) is a transdiagnostic, mindfulness-based method that seeks nondual, whole-body energy investment. Compared to ACT, BEHI is a novel, mindfulness-centered, transdiagnostic intervention aimed at fostering whole-body energy alignment and nondual awareness. By emphasizing bodily integrity, narrative coherence, and the interplay between mind and body, BEHI prioritizes long-term psychological and physical resilience. Its principles are rooted in biosemiotics and embodied cognition, offering an alternative pathway to health improvement.^20–22^ For patients with IBD, BEHI seeks to mitigate the burdens of stress, maladaptive coping, and energy dysregulation by employing body-centered techniques such as Qigong and bioenergetic exercises.^23^ Studies have highlighted BEHI’s potential for improving psychological and physical outcomes across diverse conditions, including chronic pain and post-traumatic stress.^24^ By promoting holistic healing and reducing the cognitive and emotional load of maladaptive patterns, BEHI offers a tailored strategy for addressing the multidimensional challenges faced by patients with IBD.

In this study, we decided to investigate the effects of ACT and BEHI on IBD disease because of the similarities and differences between these 2 approaches. ACT tries to forecast and influence the ongoing interaction between the entire and the particular situations, maintains the connection between the psychological event and its situation, and analyzes psychological events to preserve its integrity.^25^ However, in addition to maintaining psychological integrity, BEHI integrates hedonic and eudaimonic aspects of well-being by utilizing body-centered techniques like Qigong and bioenergetic exercises grounded in biosemiotics and embodied cognition.^20,21^ Both approaches, BEHI and ACT, are contextual and non-pathological and have incorporated cognitive, behavioral, and mindful therapeutic modalities. Therefore, this study investigates the effectiveness of a BEHI program^23^ versus an ACT program on perceived stress, quality of life, sense of coherence, and personality type D in patients with IBD.

## Methods

### Design Study and Participant

This study used a quasi-experimental method with a pre-test, post-test, and follow-up design with a control group. The research population included all women and men with IBD referred to an internist in 2023 in Isfahan. A total of 37 patients were randomly selected based on a random allocation system. We used a randomized number list generated by a random list generator software provided by a collaborating team of statisticians at the Hamedan University of Medical Science, Faculty of Pharmacy. Thirty-seven subjects were randomly assigned into 2 experimental and one control group. The first experimental group (*n* = 12) underwent ACT within eight 90-minute weekly sessions, which lasted eight weeks. The second group (*n* = 12) experienced bio-energy economy within eight 90-minute weekly sessions, again lasting for eight weeks. The control group (*n* = 13) received no training in this period.

At last, the post-test was carried out on the 3 groups. These groups were given the follow-up test after one month of education, and the effectiveness of the independent variable on tests and the stability of the teaching effect was investigated using statistical methods. Data were collected at 3 different time points, including time 0 (baseline or pre-treatment), time 1 (after-treatment), and time 2 (4-month follow-up).

### Sample Size

Using the random table method, the participants were placed into 2 groups (control and experimental). The sample size was determined using G*Power software at a significance level of 0.05, test power of 0.90, and effect size of 1.42.

### Instruments and Variable

#### WHOQOL-BREF (Measuring Quality of Life)

To measure the health-related quality of life of ESRD (End-Stage Renal Diseas) patients, the WHOQOL group created the World Health Organization Quality of Life-BREF (WHOQOL-BREF) generic health questionnaire. 1167 residents of Tehran were studied to confirm the validity and reliability of this questionnaire. Two groups of participants, one for chronic diseases and the other for non-chronic ones, were formed. The WHOQOL-BREF tool was validated in a study conducted in Tehran involving 2 groups: one comprising individuals with chronic illnesses (specific types of chronic illnesses were not detailed) and the other including individuals without chronic health conditions. The internal consistency of the tool, measured using Crohnbach’s alpha, demonstrated acceptable to good reliability across its subscales: environmental health (0.84), social relations (0.75), mental health (0.77), and physical health (0.77).^26^ This indicates the tool’s suitability for assessing quality of life in both chronic and non-chronic populations.

#### The sense of coherence

Flensburg’s Sense of Coherence (SOC) questionnaire is a 35-question test. The minimum and maximum scores that the participants get from this scale will be 35 and 105, respectively. The reliability of this questionnaire has been reported as 0.87 in one study and 0.86 in another. In another research, the reliability of this questionnaire to Cronbach’s alpha method was 0.84.^27^

#### Type D personality questionnaire

Denollet developed this scale in 2005 to quantify the elements of social inhibition and negative affect. The lowest and greatest scores on this scale are 14 and 56, respectively. For negative affect and social inhibition, the test-retest reliability coefficients are 0.87 and 0.82, respectively.^28^ Additionally, internal investigations have revealed that the psychometric qualities of this questionnaire are appropriate and positive.^29^

#### Perceived stress scale

The creators of the perceived stress measure were Cohen.^30^ According to Cohen’s calculations, the Perceived Stress Scale (PSS) has a test–retest reliability of 0.85 and an internal consistency estimate between 0.84 and 0.86. Maroufizadeh, Zareiyan, and Sigari^31^ have confirmed the similarity coefficient of the questionnaire’s items in the Iranian population, and the items’ Cronbach’s alpha has been determined to be 0.84.

### Acceptance and Commitment Therapy

The ACT program was 6 weekly sessions for shaping ways of limiting the influence of stressful thoughts and feelings on day-to-day living and goal achievement by increasing psychological flexibility. Group-based intervention with 8 weekly sessions, each lasting 90 minutes. Sessions were conducted by a trained psychologist experienced in ACT techniques.

### Bioenergy Economy-based Health Improvement

The Bioenergy Economy-based Health Improvement (BEHI) intervention was based on a structured psycho-educational program adapted from Goli’s BEHI model.^32^ In this study, the BEHI program was a 90-minute program and was conducted weekly for 6 consecutive weeks in a group-based intervention. Sessions were led by a trained facilitator knowledgeable in BEHI techniques and body-centered therapies. The podcast was played for the group in each session in the hospital, and patients were asked to perform the same exercises performed in each session along with the presentation twice a week. Each podcast includes lessons, exercises, lesson summaries, and weekly assignments. It is worth noting that all the patients of the case group joined a telegram group, and were presented with the exercise file (in more detail) as a separate file after each session. This file consisted of a 30-40-minute audio recording. They were also asked to report on the implementation of the assignments and their number in a form and submit it on the next session. The structure of the first session differed from that of the following sessions, and included introductions, and familiarization with and preparation for the course. Then, 90 minutes of listening to the podcast and performing the exercises began under the supervision of a trained facilitator.

Subsequent weekly sessions began with 30 minutes of feedback on weekly exercises and physical, mental, and communication changes, as well as answers to the participants’ questions. Then, the first part of the podcast (lesson) was presented for 45 minutes, and after a 30-minute rest and break, the second part of the podcast (presenting the exercises) was presented for 45 minutes, and finally, the session ended with 30 minutes of feedback on exercises, answering questions, and presenting assignments. No specific intervention was performed in the control group, although both groups (case and control) received routine cardiovascular medications and routine care. The summary of sessions is illustrated in Table 1.

**Table 1.** Summary of BEHI session

Session	Topic	Subject	Exercise
1	Relaxation	Work-burden/mind-body coordination, stress response/release	Abdominal breathing/gradual relaxation/body purification
2	Tensegrity	Somatic memory, armor/integrity-safety	Vibration/tensegrity exercises
3	Body awareness	Body sense, salutogenesis	Body awareness (superficial, deep, balanced, and visceral senses)
4	Attention work	Attention skewness/conscious direction of attention, danger brain-communication brain/gratitude	Attention/gratitude exercises, Bioenergy work
5	Narrative work	Narrative skewness (resentment/blame/greed/melancholia), non-life/self-care bias, time and narration (memory reconstruction)/narration and body tune	Body caress, lack of interpretation, pragmatic speech, body awareness
6	Relation work	Relation-nature/selves/avoidance of rejection/limit and love/In-field and synergy/relational body	Positive no/sharing, biofield work
7	Liberation from non-life (forgiveness: inter/intrapersonal)	Death instinct?!/Repetition fate/stabilized anger/why we do not forgive/value bias/body bias	Biofield work/refining resentments (forgiveness with guided imagination), body purification
8	Path of love (forgiveness: transpersonal)	Transpersonal dimension/openness to whole/unconditioned health providing/kindness: mature defense/submission/intentional force	Wholeheartedness, love meditation (transpersonal forgiveness)

### Analysis

Statistical analyses were performed using the Perceived Stress Scale (PSS) version 15 (IBM Corp, Armonk, NY). Quantitative and qualitative data were expressed as mean ± standard deviation (SD) and percentage, respectively. To apply the mixed variance analysis method, the presumption of normality of the sample distribution of the scores of the sample groups was obtained from the Shapiro-Wilk and Kolmogorov-Smirnov tests. In this research, mixed analysis of variance was used for the statistical analysis of the data, and Levene’s test was used to check the assumption of equality of variances. Also, Mauchly’s test was used to check the assumption of Sphericity, which is mixed with the assumptions of variance analysis.

### Ethics

Ethical considerations (Ethical code: IR.IAU.NAJAFABAD.REC.1398.120) in this research were such that participation in this research was completely voluntary. Before starting the project, the participants were familiarized with the specifications of the project and its regulations.

## Results

In this study, 37 individuals with a mean age of 36.15 ± 2.77 years for the experimental group based on the Bioenergy Economy approach, 37.75 ± 2.13 years for the experimental group based on the ACT approach, and 35.90 ± 2.26 years for the control group participated. In the experimental group based on the Bioenergy Economy, 7 people (equivalent to 58.33%) were women, and 5 people (equal to 41.67%) were men. In the experimental group based on Acceptance and Commitment Therapy, 8 people (equivalent to 66.67%) were women, and 4 (equal to 33.33%) were men. In the control group, 8 people (61.54%) were women, and 5 (equivalent to 38.46%) were men.

According to Table 2, the mean scores of the perceived stress and D personality type decreased, and the mean scores of the sense of coherence and quality of life increased in the post-test and follow-up of the ACT and BEHI groups. To investigate the effectiveness of treatment based on BEHI and treatment based on ACT on perceived stress, quality of life, sense of coherence, and D personality type in patients with IBD. First, the effectiveness of each of the independent variables (Bioenergy Economy-based Health Improvement and acceptance and commitment therapy) was tested separately on the perceived stress, quality of life, sense of coherence, and D personality type of patients with IBD, and then, if these 2 interventions are effective, the effectiveness of these interventions should be compared. The results of Mauchly’s test show that the assumption of Sphericity, an assumption of mixed variance analysis, is fulfilled for all dependent variables.

**Table 2.** Mean and standard deviation of pre-test, post-test, and follow-up scores of the research variables

Variables	Groups	Pre-test		Post-test		Follow-up	
		Mean	SD	Mean	SD	Mean	SD
Perceived stress	BEHI	32.00	9.26	24.75	7.87	26.00	7.85
	ACT	34.91	6.06	28.33	5.43	29.00	5.35
	Control	36.00	7.21	37.00	7.42	36.61	7.42
Quality of life	BEHI	70.66	8.34	87.41	5.48	84.75	5.84
	ACT	62.25	8.36	71.58	10.66	69.66	9.51
	Control	59.69	5.03	57.07	5.88	58.77	4.85
Sense of coherence	BEHI	57.00	6.04	65.58	6.65	64.50	5.69
	ACT	56.08	5.20	63.58	5.31	62.66	5.75
	Control	56.23	5.90	53.53	5.51	54.84	5.47
D personality type	BEHI	36.83	6.24	30.3	7.24	31.41	6.44
	ACT	36.33	4.93	29.66	4.09	30.58	4.14
	Control	35.84	5.59	36.77	6.12	36.69	6.07

Table 3 results indicate that the interaction effect of the type of treatment (Bioenergy Economy-based Health Improvement or acceptance and commitment therapy) and the time factor on the perceived stress, quality of life, sense of coherence, and D personality type scores of patients with IBD are significant (*P* < .001). Therefore, it is concluded that the type of treatment received (Bioenergy Economy-based Health Improvement or acceptance and commitment therapy) significantly affected these variables in patients with IBD in different evaluation stages. There is a significant difference between the pre-test, post-test, and follow-up mean scores in the perceived stress, quality of life, sense of coherence, and D personality type variables. This means that the Bioenergy Economy-based Health Improvement and acceptance and commitment therapy have significantly changed the post-test scores and follow-up of the perceived stress, quality of life, sense of coherence, and D personality type compared to the pre-test stage. Another finding of this table showed that there is no significant difference between the average scores of the post-test and follow-up stages. This finding can be explained by the fact that the perceived stress, quality of life, sense of coherence, and D personality type scores of IBD patients, which significantly decreased in the post-test, could maintain these changes during the follow-up period.

**Table 3.** The results of mixed variance analysis

Variables	Source	SS	Df	MS	*F*	*P*-value	Eta
Perceived stress	Time	93.95	1.60	58.71	152.16	0.001	0.84
	Time * group	42.75	1.60	26.71	69.24	0.001	0.71
	Group	157.34	1	157.34	52.34	0.001	0.55
Quality of life	Time	1329.80	1.02	664.90	72.69	0.001	0.72
	Time * group	1243.35	1.02	1211.36	67.97	0.001	0.70
	Group	4869.37	1	4869.37	55.81	0.001	0.66
Sense of coherence	Time	159.01	1.38	115.04	147.03	0.001	0.76
	Time * group	90.98	2.76	32.91	42.01	0.001	0.65
	Group	1012.34	2	506.17	40.30	0.001	0.64
D personality type	Time	43.28	2	21.64	153.21	0.001	0.84
	Time * group	39.46	2	17.73	139.68	0.001	0.83
	Group	44.10	1	44.10	43.53	0.001	0.44

Table 4 results showed that there were significant differences between the effectiveness of Bioenergy Economy-based Health Improvement and acceptance and commitment therapy to the perceived stress and quality of life of patients with IBD. In this way, the Bioenergy Economy-based Health Improvement had a more significant impact on the perceived stress and quality of life of patients with IBD than the acceptance and commitment therapy. However, there was no significant difference between the effects of these 2 treatments for the sense of coherence and D personality type factors.

**Table 4.** Pairwise comparison using the Bonferroni post hoc test

Variables	Group (I)	Group (J)	Mean Diff.	*P*
Perceived stress	BEHI	ACT	−3.16	.02
		Control	−8.95	.001
	ACT	BEHI	3.16	.02
		Control	−5.78	.01
Quality of life	BEHI	ACT	13.11	.001
		Control	22.43	.001
	ACT	BEHI	−13.11	.001
		Control	9.32	.001
Sense of coherence	BEHI	ACT	1.58	.74
		Control	7.48	.001
	ACT	BEHI	−1.58	.74
		Control	5.90	.001
D personality type	BEHI	ACT	0.66	.69
		Control	−3.57	.001
	ACT	BEHI	−0.66	.69
		Control	−4.24	.001

We can conclude that there was a significant difference between the experimental groups and the control group over time (*P* < .01). This indicates that the interventions had a notable effect, with both ACT and BEHI showing improvements in perceived stress, quality of life, sense of coherence, and Type D personality. However, the BEHI program demonstrated a greater overall impact in reducing perceived stress and Type D personality, as well as enhancing the quality of life, compared to the ACT program.

## Discussion

This research aims to compare the effectiveness of ACT and BEHI on perceived stress, quality of life, sense of coherence, and D personality type in patients with IBD. Results revealed that perceived stress, quality of life, sense of coherence, and D personally type significantly improved in the post-test and follow-up stages through the ACT and BEHI programs. It was found that there was a significant difference between the experimental groups and the control group over time. According to the results, the BEHI program was more effective in decreasing perceived stress and D personality type and increasing quality of life than the ACT program. Although the BEHI program improved the sense of coherence, the ACT program was more effective in increasing the sense of coherence in patients.

The sense of coherence refers to the extent to which individuals perceive their lives as comprehensible, manageable, and meaningful. An increase in the sense of coherence indicates that patients feel more in control and capable of managing their illness, leading to better psychological and emotional outcomes.^33^

Likewise, other scientific studies based on ACT and BEHI approaches investigate the effects of these 2 programs on psychological factors. While some of these studies support the results of the current study, a few findings disapprove of ours. The ACT conducted by Wynne^34^ improved stress and other indices of psychological health in IBD patients, which aligns with this study’s findings. In another study in 2022,^35^ the ACT for IBD program was conducted on patients with IDB. The findings of this meta-analysis also highlight the need for trials of psychotherapy interventions explicitly targeting the groups at risk of suboptimal outcomes (eg, people with psychological distress) to improve both mental and physical health management in IBD. However, Lavelle and his colleagues^18^ mentioned that ACT can have adverse effects on some participants and took an opposite view. This study explained that despite 40% and 58% of participants experiencing improvements across various stress and psychological flexibility outcomes, many participants experienced minimal change or deterioration following exposure to the ACT intervention. For some participants, deterioration occurred due to poorer adherence or not having engaged in all intervention components. Regarding the absence of changes, we note that in some cases, the change may have been obfuscated by that participant’s dropout.

Besides the profound effects of the ACT program on the psychological factors of patients with IBD in this study, it seems that the BEHI program might offer a more holistic and cohesive approach for patients with IBD. BEHI can be an effective program for body self-concept.^36^ Previous studies based on the BEHI program are in line with the findings of the current results and have previously been employed for improving psychological factors and physical symptoms of patients in different medical and psychological conditions such as post-traumatic stress,^24^ breast cancer,^37^ high anxiety sensitivity,^38^ Obesity,^36^ coronary heart disease,^39^ Myofascial Pain Syndrome,^36^ Tethered Cord Syndrome,^40^ and Migraine Headache.^41^

Cultivating bodily awareness and adopting a mindful perspective deters individuals from being overly fused with and hyper-identified with their thoughts.^21^ This bodily awareness can reduce anxiety and depression and enhance QQL significantly.^42^ BEHI prioritizes the alignment and synchronization of energy allocations toward fostering well-being by reducing the bodily load of nonfunctional thoughts, habits, dissonances, and ambivalences.^43^ As such, BEHI assesses and signifies the potential impact of a shift in mindset and mind-body coordination on distress. If an individual’s prevailing mental framework perceives life as a collection of isolated fragments and insists that each part must be perfect for the entirety to be correct, achieving unity becomes elusive. By transitioning this perspective towards wholeness and cultivating an upward-down (top-down) organization, BEHI facilitates personal health and healing. Initially, embracing a sense of incompleteness and experiencing wholeness can prove advantageous, allowing the healing process to unfold naturally. In this context, one may realize that separating individual components isn’t essential to attain wholeness. Wholeness signifies an embrace of the entire entity rather than the precision of each part in isolation.^44^ These aspects of wholeness, the promotion of well-being, and the concept of boundlessness receive less emphasis in ACT. The concept of intentionality and shifts in healing expectations are crucial in enhancing the therapeutic meaning response, a central focus within the BEHI program.^23^

The current study faces a limitation regarding the generalizability of its findings. Although well-powered in terms of methodology and featuring a relatively large number of participants and data points, the study samples are still relatively small and homogenous. While the meta-analysis of effects across participants contributes to some generalizability, conducting more extensive studies and additional replications is essential to enhance overall generalizability. Another limitation was the absence of screening for existing psychological diagnoses and the utilization of psychological support. While no participants openly shared information about pre-existing conditions or ongoing psychological therapy, there is a possibility that some chose not to disclose such details. Consequently, the effectiveness or ineffectiveness of the interventions could be influenced by concurrent therapies and undisclosed mental health conditions.

Future studies should consider the potential impact of IBD subtypes (Crohn’s disease vs. ulcerative colitis) and the severity of the disease on the effectiveness of the interventions.

## Conclusion

In summary, the integration of either the ACT or BEHI programs into biomedical therapies for individuals with IBD has the potential to mitigate perceived stress and D personality type while also improving the sense of coherence and quality of life through the facilitation of psychological and lifestyle adjustments. While both interventions demonstrated notable efficacy in improving patients’ psychological factors, the BEHI program exhibited a heightened level of significance in moderating mental manifestations in individuals with IBD.

## Data Availability

No new data were created or analyzed in this study. Data sharing is not applicable to this article as no datasets were generated or used during the current study.

## Notes

Summary of the article’s main point: The main point of this study is to compare BEHI and ACT approaches in perceived stress, quality of life, sense of coherence, and D personality type in patients with IBD.
